# Macrophages in lung fibrosis

**DOI:** 10.1093/intimm/dxab040

**Published:** 2021-07-16

**Authors:** Tatsuro Ogawa, Shigeyuki Shichino, Satoshi Ueha, Kouji Matsushima

**Affiliations:** Division of Molecular Regulation of Inflammatory and Immune Diseases, Research Institute for Biomedical Sciences, Tokyo University of Science, 2669 Yamazaki, Noda, Chiba 278-0022, Japan

**Keywords:** chemokine, fibroblasts, growth factor, interstitial lung disease, single-cell transcriptome

## Abstract

Pulmonary fibrosis (PF) is a disease in which excessive extracellular matrix (ECM) accumulation occurs in the lungs, which induces thickening of the alveolar walls, ultimately leading to the destruction of alveolar structures and respiratory failure. Idiopathic PF, the cause of which is unknown, has a poor prognosis with a median survival of 2–4 years after diagnosis. There is currently no known curative treatment. The mechanism underlying PF is thought to be initiated by the dysfunction of type II alveolar epithelial cells, which leads to ECM overproduction through the activation of fibroblasts. In addition, it has been suggested that a variety of cells contribute to fibrotic processes. In particular, clinical and basic research findings examining the roles of macrophages suggest that they may be pivotal regulators of PF. In this review, we discuss the characteristics, functions and origins of subsets of macrophages involved in PF, including resident alveolar, interstitial and monocyte-derived macrophages.

## Introduction

Pulmonary fibrosis (PF) (PF) is a disease in which the accumulation and activation of fibroblasts lead to disruption of the normal lung structure through excessive deposition of extracellular matrix (ECM) components such as collagen. PF is a type of interstitial lung disease (ILD) in which the interstitium of the lung is the main site of inflammation and fibrosis. ILDs are classified into two groups: those with known causes, such as connective tissue disease-related ILD (CTD-ILD), drug-induced ILD (DILD), pneumoconiosis, sarcoidosis and chronic hypersensitivity pneumonitis; and those with unknown causes, idiopathic interstitial pneumonias (IIPs) (1). IIPs are further classified into various subtypes based on imaging and pathological diagnosis, the most common of which is idiopathic PF (IPF), which has a poor prognosis with a median survival of 2–4 years after diagnosis (2, 3).

IPF is characterized by severe fibrosis and the formation of a honeycomb lung, resulting in restrictive ventilation and impaired lung diffusion capacity. One histological feature of IPF is a usual interstitial pneumonia (UIP) pattern characterized by spatially and temporally heterogeneous and diverse fibrotic lesions and fibroblastic foci where collagen, fibroblasts and myofibroblasts accumulate in the interstitium. Chronic inflammation and repeated injury to alveolar epithelial cells are considered causes of fibrosis and scarring. However, the relative lack of inflammatory features in IPF lungs and the ineffectiveness of anti-inflammatory and immunosuppressive drugs challenge this concept (4).

Accumulating evidence indicates that the dysfunction of alveolar epithelial cells, especially type II alveolar epithelial cells (AT2), is the starting point for the development of fibrosis. This is supported by the fact that AT2-specific gene mutations cause spontaneous PF in humans and mice, and AT2 hyperplasia has been observed adjacent to the honeycomb region in IPF lungs (5). In addition, macrophages are attracting attention as regulators of pathogenic fibrotic responses because they localize close to myofibroblasts, which are considered the major source of ECM in PF and express high levels of fibroblast-activating factors (6). Furthermore, elevated expression of macrophage-derived molecules is observed in IPF lungs (7). In this review, we will focus on macrophages and describe them in detail. The relative contribution of the acquired immune system is controversial because fibrosis develops and progresses in mice lacking acquired immunity (8, 9).

Although the importance of epithelial cells and macrophages in IPF has previously been highlighted, much remains unknown and there are no therapeutic agents that target these cells. Therefore, the two currently approved IPF drugs mainly target fibroblasts. One is pirfenidone, which inhibits the production of pro-inflammatory cytokines (TNF-α, IL-1, IL-6, etc.) and promotes the production of anti-inflammatory cytokines (IL-10) while preserving the anti-fibrotic cytokine IFN-γ (10). In addition, pirfenidone suppresses fibroblast proliferation and collagen production by inhibiting the production of growth factors such as transforming growth factor beta (TGF-β), fibroblast growth factor (FGF) and platelet-derived growth factor (PDGF). It is through these multiple mechanisms that pirfenidone is thought to exert its anti-fibrotic effect. The other IPF drug is nintedanib, a small-molecule tyrosine kinase inhibitor that limits fibroblast migration and proliferation by inhibiting the signaling of growth factor receptors including PDGF receptors (PDGFRα and PDGFRβ), FGF receptors (FGFR1, FGFR2 and FGFR3), and vascular endothelial growth factor receptors (VEGFRs) (11).

However, the effects of these drugs are limited to delaying disease progression. Thus, it is necessary to clarify the molecular and cellular mechanisms involved in disease progression and identify targets for intervention to terminate or reverse disease progression.

## Classification of macrophages in lung tissue

Macrophages in the lungs are classified as either alveolar macrophages (AMs) or interstitial macrophages (IMs) on the basis of their localization. Resident AMs in normal mouse lungs characteristically express high levels of SiglecF, CD169 and CD206. They are maintained by granulocyte macrophage colony-stimulating factor (GM-CSF)-dependent activation of the nuclear receptor peroxisome proliferator-activated receptor gamma (PPAR-γ) and TGF-β stimulation (12, 13). Resident AMs have been reported to remove microbes and dead cells, catabolize surfactant molecules, induce inflammatory and immune responses triggered by foreign substances or immune tolerance via TGF-β and play a role in alveolar regeneration via amphiregulin. Indeed, dysfunction of resident AMs because of a deficiency in GM-CSF receptor (GM-CSFR)/PPAR-γ signaling results in delayed microbial clearance and alveolar proteinosis due to accumulation of surfactant molecules.

**Fig. 1.**
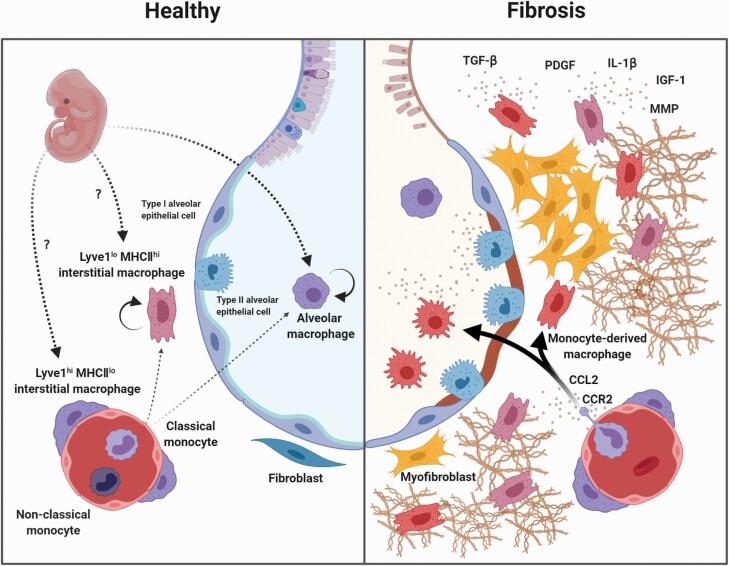
Composition and localization of macrophages in normal and fibrotic lungs in mice. There are two distinct groups of tissue macrophages in the normal lung: alveolar and interstitial. Both are capable of self-renewal but are slowly replaced by monocyte-derived macrophages. Furthermore, it has been reported that there are two subsets of interstitial macrophages: Lyve1^hi^ MHCII^lo^ interstitial macrophages are localized in the perivascular area, whereas Lyve1^lo^ MHC II^hi^ interstitial macrophages are found in the alveolar interstitium. In the fibrotic lungs, classical monocytes infiltrate in a CCR2-dependent manner and differentiate based on the specific tissue environment. However, it is worth noting that there are conflicting reports on the roles of these cells in fibrosis. Lyve1^lo^ MHC II^hi^ interstitial macrophages have also been shown to increase during fibrosis.

Macrophages are also classified as either tissue-resident or monocyte-derived. Tissue-resident macrophages are derived from fetal liver or yolk sac-derived cells and are locally maintained by self-renewal (14–16). The second group of macrophages is derived from monocytes that infiltrate the lung and differentiate there. Recently, Liu *et al*. (17) identified the *Ms4a3* gene as a granulocyte-monocyte progenitor (GMP)-specific gene by single-cell transcriptome (SCT) technology and developed a fate-mapping system labeled with the *Ms4a3* gene. Using this system, these researchers demonstrated that resident AMs are slowly replaced by monocytes in the long term (17). These monocyte-derived AMs acquire similar functions and self-renewal capacity to fetal liver or yolk sac-derived AMs in a tissue environment-dependent manner (16, 18) (Fig. 1).

IMs have been less studied than AMs. This is due in part to the low number of cells, the inability to collect these cells from bronchoalveolar lavage fluid (BALF), difficulty in discriminating between IMs and dendritic cells (DCs) by flow cytometry (FCM) because of limited surface markers, and the lack of an IM-specific intervention system. However, recent developments in SCT technology have made it possible to clearly discriminate between AMs, IMs and DCs. Furthermore, multiple subsets of IMs in normal mouse lungs have been identified using SCT (19–21). Chakarov *et al*. (20) reported on Lyve1^hi^ MHCII^lo^ IMs and Lyve1^lo^ MHCII^hi^ IMs through SCT analysis. Lyve1^hi^ MHCII^lo^ IMs are localized adjacent to blood vessels and have a high level of expression of genes related to the positive regulation of leukocyte chemotaxis, response to wounding and receptor-mediated endocytosis (20, 21).

Lyve1^lo^ MHC II^hi^ IMs are localized adjacent to nerve fibers or in the alveolar interstitium, with a high level of expression of genes related to antigen processing and presentation, regulation of T-cell activation and defense responses. Furthermore, the results of experiments using lineage tracing, parabiosis models and gene-modified mice suggest that these IMs are capable of self-renewal but, like AMs, are replaced by monocyte-derived macrophages in the long term (21) (Fig. 1).

During infection, inflammation and fibrosis, monocytes infiltrate tissues and differentiate into macrophages through multiple steps in an inflammation-dependent manner. As a result, a heterogeneous macrophage population is formed. There are two types of monocytes present in the circulation: Ly6C^hi^ CCR2^hi^ monocytes (classical monocytes) and Ly6C^lo^ CCR2^lo^ monocytes (non-classical monocytes).

It was generally believed that only classical monocytes infiltrate the lungs in a CCR2-dependent manner and differentiate into monocyte-derived macrophages, but recent findings have challenged this concept. Sabatel *et al*. (22) reported that the increase in IMs induced by CpG administration was caused by CCR2-independent infiltration of splenic reservoir monocytes, but the ligand–receptor pair responsible for the increase in IMs was not identified. In the short term, monocyte-derived macrophages have a different gene expression pattern from AMs or IMs, and their numbers decrease with the resolution of inflammation (23) (Fig. 1). In contrast, Misharin *et al*. (24) reported that some monocyte-derived macrophages can persist for a substantial amount of time, but it is not clear how these selections are determined. The relative contribution of CCR2-dependent/independent monocytes to the macrophage pool in the lung remains to be established due to the lack of a specific intervention system against CCR2^lo^ monocytes. SCT data on CCR2^lo^-specific marker genes may be helpful in addressing this issue.

Although the M1/M2 paradigm has been widely used as a functional classification of these monocyte-derived macrophages, the existence of populations expressing both markers suggests that it is difficult to classify these highly heterogeneous macrophage populations as a strict dichotomy (24). Human AMs, IMs and monocytes are CD14^lo^ CD16^+^ CD206^hi^ CD169^+^, CD14^+^ CD16^+^ CD206^+^ CD169^lo^ and CD206^–^ CD169^–^ cells, respectively. Human counterparts of murine IM subsets have also been identified as populations with similar gene expression patterns in SCT analysis (20). However, it is unclear whether these cells have the same characteristics and functions. In addition, given that the human lung is subjected to years of exposure to foreign bodies and inflammation, which is critically distinct from mice maintained in a specific-pathogen-free environment, the composition and transition of tissue-resident and monocyte-derived macrophages may be different between humans and mice.

## Role of macrophages in PF

### Macrophage-derived secretory proteins and PF

Macrophages are a major source of TGF-β, a primary effector molecule in fibrosis. They also promote fibroblast proliferation and collagen synthesis through the production of growth factors such as FGF, PDGF, VEGF and insulin-like growth factor 1 (IGF-1) (25–27). Macrophage-derived IL-1β and CCL18 have been reported to exacerbate fibrosis (6, 27). In contrast, macrophages also produce matrix metalloproteinases (MMPs) that degrade ECM, which may have an inhibitory effect on fibrosis (27). Furthermore, there are several subfamilies of MMPs, some of which promote fibrosis; therefore, it is difficult to make a general statement about these enzymes (28). For example, PF has also been reported to be suppressed in MMP12-deficient mice (29). These various, and sometimes conflicting, macrophage roles may be associated with functional skewing by distinct inflammatory microenvironments or with differences in the composition of macrophage subpopulations being related to distinct functions. Therefore, the identification of the role of each macrophage population has become a focus of PF research.

### Role of macrophages in mouse models of PF

Gibbons *et al*. (30) reported that depletion of macrophages using liposomal clodronate during the fibrotic phase in a mouse model of bleomycin-induced PF markedly suppressed pathology, suggesting the pro-fibrotic role of macrophages in fibrosis. On the other hand, they also reported that macrophage depletion in the resolution phase of bleomycin-induced PF delays this resolution, suggesting that macrophage contribution to the pathology of bleomycin-induced PF is context-dependent (30).

Furthermore, the roles of resident alveolar, interstitial and monocyte-derived macrophages in fibrosis are current areas of analysis. Misharin *et al*. (24) showed that the depletion of resident AMs by intra-tracheal administration of liposomal clodronate did not affect bleomycin-induced PF. Comparatively, experiments using *CD11c*^Cre^*Casp8*^fl/fl^ and *LysM*^Cre^*Casp8*^fl/fl^ mice revealed that depletion of monocyte-derived AMs suppresses PF. McCubbrey *et al*. (31) showed that PF was suppressed by depletion of SiglecF^lo^ CD11b^hi^ macrophages using hCD68rtTA/TetOn-Cre/*cFLIP*^fl/fl^ mice. Experiments using CCR2-deficient mice, in which CCR2-dependent monocyte infiltration is impaired, have also been reported to show reduced PF (32–34). Conversely, Liang *et al*. (35) reported that bleomycin-induced PF was suppressed in mice overexpressing CCL2, a ligand for CCR2. It has also been found that CCR2 deficiency in a mouse model of silica-induced PF expanded the fibrotic area and caused a sustained increase in fibrosis-related gene expression, suggesting a suppressive role of CCR2^hi^ monocyte-derived macrophages in fibrosis (36) (Table 1).

**Table 1.** Findings reported from models of PF

PF model	Depletion method	Depletion phase	Affected cell populations	Change in fibrosis pathology	Reference
Bleomycin	Liposomal clodronate intra-tracheally	Fibrotic phase	N.D.	Suppression	(30)
TGF-β	Liposomal clodronate intra-tracheally	Fibrotic phase	N.D.	Suppression	
Bleomycin	Liposomal clodronate intra-tracheally	Resolution phase	N.D.	Exacerbation	
Bleomycin	Liposomal clodronate intra-peritoneally	Fibrotic phase	Monocytes↓	Suppression	
TGF-β	Liposomal clodronate intra-peritoneally	Fibrotic phase	Monocytes↓	Suppression	
Bleomycin	Liposomal clodronate intra-tracheally	All phases	Resident AMs↓	No change	(24)
Bleomycin	*CD11c* ^Cre^ *Casp8* ^flox/flox^ mice	All phases	SiglecF^lo^ monocyte-derived AMs↓	Suppression	
Bleomycin	*LysM* ^Cre^ *Casp8* ^flox/flox^ mice	All phases	SiglecF^lo^ monocyte-derived AMs↓	Suppression	
Bleomycin	hCD68rtTA/TetOn-Cre/*cFLIP*^fl/fl^ mice	Fibrotic phase	SiglecF^lo^ CD11b^hi^ macrophages↓	Suppression	(31)
Bleomycin	CCR2^−/−^ mice	All phases	N.D.	Suppression	(32)
FITC	CCR2^−/−^ mice	All phases	N.D.	Suppression	
Bleomycin	CCR2^−/−^ mice	All phases	N.D.	Suppression	(33)
Bleomycin	CCR2^−/−^ mice	All phases	N.D.	Suppression	(34)
Bleomycin	CCL2 transgenic mice	All phases	Resident AMs↑, CD11b^+^ macrophages↑ DCs↑, neutrophils↓, lymphocytes↑	Suppression	(35)
Silica	CCR2^−/−^ mice	All phases	Ly6C^hi^ monocytes↓, Ly6C^lo^ monocytes↓ MHC II^+^ macrophages↓, CD11b^+^ DCs↓	Exacerbation	(36)
Asbestos	*CD11c* ^Cre^ *Casp8* ^flox/flox^ mice	All phases	SiglecF^lo^ monocyte-derived AMs↓	Suppression	(38)
Asbestos	*LysM* ^Cre^ *Casp8* ^flox/flox^ mice	All phases	SiglecF^lo^ monocyte-derived AMs↓	Suppression	
Asbestos	Pharmacological blockade of M-CSFR (Csf1r)	Fibrotic phase	Monocytes↓, monocyte-derived AMs↓, IMs↓	Suppression	
Bleomycin	*Cx3cr1*-CreERT2/Rosa26-diphtheria toxin A mice	Fibrotic phase	Mertk^+^ SiglecF^+^ macrophages↓	Suppression	(39)
Bleomycin	*Lyve1* ^Cre^/*Slco2b1*^flox/DTR^ mice	All phases	Lyve1^hi^ MHC II^lo^ IMs↓, neutrophils↑, monocytes↑ DCs↑, SiglecF^−^ Mertk^+^ Cd64^+^ macrophages↑	Exacerbation	(20)

FITC, fluorescein isothiocyanate; N.D., not determined.

These conflicting results may be due to differences in PF models, timing of intervention and specificity of treatment. In particular, the administration of liposomal clodronate induces inflammation by itself and when administered intravenously it removes populations involved in the maintenance of homeostasis throughout the body, such as Kupffer cells and red-pulp macrophages. Given that clinical trials of monocyte-targeted IPF therapeutics are already underway, clarifying the role of monocyte-derived macrophages in progressive fibrosis in humans seems to be a critical issue (37).

SCT technologies are expected to resolve the controversy regarding the roles of macrophages in PF as they provide robust classifications based on the global gene expression pattern of each cell. Using the promoter of a specific marker gene identified by SCT and genetic engineering approaches, it is possible to determine the impact specific interventions will have on particular macrophage subsets in relation to PF.

There are several reports of SCT analyses in mouse models of PF. Joshi *et al*. (38) performed SCT analysis in a mouse model of asbestos-induced PF and found that the expression of genes such as *Pdgfa* and *Csf1* was high in a population of putative monocyte-derived macrophages and that subsequent *Csf1* inhibition suppressed fibrosis. Aran *et al*. (39) reported that *Ccr2*^+^*Cx3cr1*^+^ macrophage levels increased in a mouse model of bleomycin-induced PF and that fibrosis was inhibited by *Cx3cr1*^+^ cell-specific depletion. Although there are few reports on IMs, Chakarov *et al*. (20) have shown that the depletion of Lyve1^hi^ MHCII^lo^ IMs increases leukocyte infiltration and worsens fibrosis (Table 1). However, the role of Lyvel^lo^ MHCII^hi^ IMs in PF has not been elucidated, although their number increases with the progression of PF. The suppression of PF by *Csf1* inhibition and *Cx3cr1*^+^ cell depletion reported by Joshi *et al*. (38) and Aran *et al*. (39) may include the effect of the loss of IMs. Considering that *Pdgfa* is also expressed by IMs at a high level, these cells may contribute to fibrosis (38, 39).

In addition, IM localization can influence the process of PF. We have recently developed a novel transcriptome analysis method, TAS-Seq, which is highly sensitive and accurate, and has succeeded in clarifying the temporal changes in the cellular and molecular networks in a mouse model of PF (40). The analysis also identified marker genes that could discriminate subsets of lung macrophages more specifically in both steady-state and inflammatory conditions. In addition, Satoh *et al*. (41) reported that the segregated-nucleus-containing atypical monocytes (SatMs) with a bi-lobed segmented nuclear shape and Ceacam^+^ Msr1^+^ LY6C^–^ F4/80^–^ Mac1^+^ phenotype appear in a fibrosis-specific manner and contribute to the worsening of fibrosis.

### Analysis of macrophages in human IPF lungs

SCT analyses of human IPF lungs have also been reported. The fibrotic lungs are composed of a distinctly different macrophage population compared with normal lungs. Reyfman *et al*. (42) performed SCT analysis using IPF lung biopsies. They found that gene expression patterns could be classified into four macrophage populations, two of which were found to be specific to fibrotic lungs and showed high expression levels of *SPP1* and *CHI3L1* (42).

Morse *et al*. (43) performed SCT analysis of IPF lung biopsies and found that the macrophages could be divided into three populations: *FABP4*^hi^, *SPP1*^hi^ and *FCN1*^hi^ macrophages. Among them, only *SPP1*^hi^ macrophages tended to increase in fibrotic lungs (43). This study also suggested MERTK, which is highly expressed in *SPP1*^hi^ macrophages, as a potential therapeutic target for IPF. Adams *et al*. (44) reported an SCT analysis of IPF and chronic obstructive pulmonary disease (COPD) lung biopsies. Furthermore, Ayaub *et al*. (45) analyzed the data reported by Adams *et al*. (44) in detail and found that in addition to a similar increase in *SPP1*^hi^ macrophages, these macrophages have intermediate features between AMs and IMs. These studies commonly reported that *SPP1*^hi^ macrophage levels increased in IPF lungs. However, the origin of these cells and their role in either promoting or suppressing fibrosis remains unclear.

## Conclusions

In normal mouse lungs, there are resident AMs and IMs, which are slowly replaced by monocyte-derived macrophages. Experimental approaches, including SCT analyses, have shown that inflammation and fibrosis are accompanied by a transient increase in monocyte-derived macrophages and the formation of heterogeneous macrophage populations. In addition, the existence of two subsets of IMs has been demonstrated.

The role of some of these heterogeneous macrophage populations in fibrosis has also been analyzed and monocyte-derived macrophages have been relatively well studied. However, there are some conflicting results due to the differences and the limitation in the specificity of intervention approaches against monocyte-derived macrophages. In addition, it is important to clarify whether these reported monocyte-derived macrophages are differentiated from classical or non-classical monocytes, and whether they are CCR2-dependent. Analysis of IPF lung biopsies revealed a specific increase in *SPP1*^hi^ macrophages in fibrotic lungs. Future detailed studies on these macrophages are expected to establish new therapeutic strategies for IPF.

It is also worth investigating whether these macrophage populations promote or suppress fibrosis and whether they are monocyte-derived or resident macrophages that are self-renewing and activated. However, some issues need to be addressed to verify this. One is the lack of cell type-specific interventions in humans, and another is the unavailability of well-established human macrophage counterparts in mice. Even when macrophage populations with relatively similar gene expression exist, they do not always match in terms of function and localization. A new experimental system, such as a humanized mouse model (MISTIRG) expressing human cytokines and transplanted with human CD34^+^ hematopoietic stem and progenitor cells (HSPCs) and alveolar organoids, may overcome these issues, clarify the roles of various macrophages in human fibrosis and provide novel therapeutic strategies in the future (46).
